# Association between Leukocyte and Metabolic Syndrome in Urban Han Chinese: A Longitudinal Cohort Study

**DOI:** 10.1371/journal.pone.0049875

**Published:** 2012-11-27

**Authors:** Wenjia Meng, Chengqi Zhang, Qian Zhang, Xinhong Song, Haiyan Lin, Dongzhi Zhang, Yongyuan Zhang, Zhenxin Zhu, Shuo Wu, Yanxun Liu, Fang Tang, Xiaowei Yang, Fuzhong Xue

**Affiliations:** 1 Department of Epidemiology and Health Statistics, School of Public Health, Shandong University, Jinan, China; 2 Health Management Center, Shandong Provincial QianFoShan Hospital, Jinan, China; 3 Center for Health Management, Provincial Hospital Affiliated to Shandong University, Jinan, China; 4 Department of Public Health Sciences, School of Medicine, University of California Davis, Davis, California, United States of America; Brigham & Women's Hospital, and Harvard Medical School, United States of America

## Abstract

**Background:**

Although cross-sectional studies have shown that leukocyte is linked with metabolic syndrome (MetS), few longitudinal or cohort studies have been used to confirm this relationship. We therefore conducted a large-scale health check-up longitudinal cohort in urban Chinese population from middle to upper socioeconomic strata to investigate and prove the association between the total leukocyte/its subtypes and MetS/its components (obesity, hyperglycemia, dyslipidemia, and hypertension).

**Methods:**

A longitudinal cohort study was established in 2005 on individuals who were middle-to-upper class urban Chinese. Data used in this investigation was based on 6,513 participants who had at least three routine health check-ups over a period of six-year follow-up. Data analysis was conducted through generalized estimating equation (GEE) model.

**Results:**

A total of 255 cases of MetS occurred over the six-year follow-up, leading to a total incidence density of 11.45 per 1,000 person-years (255/22279 person-years). The total leukocyte was markedly associated with MetS (RR = 2.66, 95%CI = 1.81–3.90], p<0.0001) and a dose-response existed. Similar trends can be found in monocytes, lymphocytes, and neutrophils compared with the total leukocyte. The total leukocyte, neutrophil, monocyte and eosinophil levels were strong and independent risk factors to obesity, total leukocyte and neutrophil to dyslipidemia and hyperglycemia, while neither total leukocyte nor its subtypes to hypertension.

**Conclusion:**

Total leukocyte/its subtype were associated with MetS/its components (obesity, dyslipidemia and hyperglycemia), they might provide convenient and useful markers for further risk appraisal of MetS, and be the earlier biomarkers for predicting cardiovascular disease than the components of MetS.

## Introduction

Metabolic syndrome (MetS) refers to a constellation of metabolic and cardiovascular disease (CVD) risk factors, characterized by obesity, hyperglycemia, dyslipidemia, hypertension and insulin resistance, proinflammatory state, and prothrombotic state [1], [2]. It is rapidly increasing in prevalence and poses as a major challenge to public health worldwide [3]–[7]. The fact that MetS is accompanied by a low-grade inflammation suggests inflammation may play an important role in the etiology [8], [9]. Various cross-sectional studies indicated that serum C-reactive protein (CRP) levels were higher among individuals with increased MetS risk factors [10]–[14], and a large cohort study of healthy American women over an eight-year follow-up suggested that CRP had clinically important prognostic information to MetS [15]. Although high sensitivity CRP (hs-CRP) was superior to leukocyte count as an inflammatory component of MetS in Japanese [16], the latter provided a higher diagnostic accuracy for MetS in a study of Koreans, suggesting that leukocyte may be a risk and prognostic factor for the syndrome when hs-CRP is not available. In addition to cross-sectional studies with suggestions that elevated leukocyte counts were a surrogate marker for MetS [17]–[23], some attempts were also made with cohort or longitudinal data indicating that leukocyte was a potential causal factor of MetS [24]–[26].

Despite awareness of the limitation with cross-sectional studies [18]–[23], the longitudinal studies just mentioned [25], only utilized the baseline leukocyte count which is known to fluctuate during the lifespan of most people. Furthermore, few studies have assessed the relationship between subtype(s) of leukocyte and MetS/its components (obesity, hyperglycemia, dyslipidemia, and hypertension). We, therefore, conducted a large-scale health check-up based longitudinal cohort study in urban Han Chinese population from middle to upper socioeconomic strata, and the generalized estimating equation (GEE) model was used to detect the association between the total leukocyte/its subtypes and MetS/its single components. The longitudinal cohort study allowed us to use repeated observations of the same set of variables during the follow-up, and the GEE model can not only adjust for the inherent correlations between the observations, but also provide robust standard errors for regression coefficients when the independence assumption is violated [27]. Since the total l leukocyte/its subtypes are simple, readily available and inexpensive measures, findings from such a study may provide convenient and useful markers for further risk appraisal of MetS.

**Table 1:** Distribution of leukocyte/its subtype, and other potential confounding factors.

		baseline	1-yearfollow-up		2-year follow-up		3-year follow-up		4-year follow-up		5-year follow-up	
variables	N	Mean±Std	N	Mean±Std	N	Mean±Std	N	Mean±Std	N	Mean±Std	N	Mean±Std
Leukocyte	1480	6.26±1.42	3988	6.27±1.48	5260	6.26±1.54	5465	6.26±1.47*	5617	6.26±1.50	3920	6.28±1.50*
lymphocyte	1480	2.09±0.56	3988	1.87±0.50*	5260	2.04±4.76	5465	1.93±0.57	5617	2.00±0.64	3920	2.21±0.59*
monocyte	1480	0.37±0.11	3988	0.35±0.11	5260	0.33±0.11*	5465	0.33±0.12*	5617	0.34±0.13	3920	0.36±0.12
neutrophil	1480	3.64±1.14	3988	3.79±1.16*	5260	3.70±1.16	5465	3.63±1.14	5617	3.66±1.13	3920	3.64±1.15
eosnophil	1480	0.15±0.15	3988	0.13±0.13	5260	0.14±0.12	5465	0.13±0.12	5617	0.14±0.13	3920	0.14±0.12
basophil	1480	0.02±0.02	3988	0.02±0.03	5260	0.02±0.02	5465	0.02±0.01	5617	0.01±0.01	3920	0.03±0.02
age	1571	38.86±11.73	4064	39.28±11.78	5407	40.47±11.98	5612	40.78±11.72	5642	41.95±11.73	3925	43.02±11.93
GGT	1480	17.67±17.4	3988	18.30±16.16	5260	20.27±19.66*	5465	19.08±16.59*	5617	20.63±20.19*	3920	21.57±19.6*
ALB	1480	45.91±1.91	3988	47.36±2.15*	5260	45.25±2.74	5465	45.81±2.88	5617	44.88±2.62	3920	45.12±2.37
GLO	1480	27.81±3.58	3988	26.22±3.88	5260	27.30±3.90	5465	27.73±3.85	5617	28.82±3.45	3920	31.51±3.37*
BUN	1480	4.86±1.2	3988	4.82±1.19	5260	4.65±1.17	5465	4.70±1.17	5617	4.73±1.2	3920	4.82±1.15
S-Cr	1480	79.59±13.51	3988	76.36±14.2	5260	77.72±13.56	5465	78.28±14.17	5617	78.76±14.42	3920	75.49±12.49*
TC	1480	4.74±0.86	3988	4.80±0.86	5260	4.85±0.90	5465	4.84±0.93	5617	5.00±0.95	3920	5.22±0.96*
Hb	1480	142.08±15.24	3988	142.77±14.74	5260	143.67±15.13	5465	141.46±14.99	5617	142.22±15.35	3920	141.97±16.51
HCT	1480	42.9±4.03	3988	43.19±3.86	5260	42.45±3.86	5465	42.2±3.84	5617	42.02±4.00	3920	41.64±3.88
MCV	1480	90.64±4.88	3988	90.36±4.7	5260	30.32±2.08	5465	89.64±4.72	5617	89.71±4.92	3920	88.53±4.90*
MCH	1480	30.00±2.00	3988	29.85±1.91	5260	12.77±0.98	5465	30.03±2.07	5617	30.36±2.29	3920	30.15±2.16
RDW	1480	12.74±1.07	3988	12.83±0.98	5260	6.04±1.47	5465	12.78±0.96	5617	12.77±1.03	3920	12.82±1.03*
PDW	1480	12.31±1.70	3988	12.38±1.75	5260	12.31±1.71	5465	12.27±1.68	5617	12.17±1.66	3920	12.46±1.63*
MPV	1480	10.46±0.84	3988	10.45±0.99	5260	10.43±0.81	5465	10.44±0.80	5617	10.37±0.80	3920	10.47±0.80
PCT	1480	0.23±0.10	3988	0.25±0.1	5260	0.26±0.32	5465	0.25±0.05	5617	0.25±0.05	3920	0.25±0.32

* P<0.05.

The abbreviations of the variables: GGT = gamma-glutamyltranspeptidase; ALB =  serum albumin; GLO = serum globulins; BUN = blood urea nitrogen; S-Cr = serum creatinine; TC = Total cholesterol; Hb = Hemoglobin; HCT = Hematokrit; MCV = mean corpuscular volume; MCH = mean corpuscular hemoglobin; RDW =  Red blood cell distribution width; PDW = Platelet distribution width; MPV =  mean platelet volume;

PCT  = Thrombocytocrit; Diet: 0: Vegetarian, 1: normal, 2: meat-based 3: sea food (the major kinds of food used to have); Drinking: 0: never, 1: seldom, 2: often, wine,3: often beer, 4: often, Chinese spirits,5: often, mixed all kinds; Smoking: 0: never,1: seldom,2: quit,3:1–4/d, 4: 5–15/d, 5: >15/d; Quality of sleep 0: excellent, 1: well, 2: fair 3: poor, 4: very poor (evaluated by themselves); Physical activity 0: never, 1: seldom (1–2 times a week), 2: often or everyday (more than 3 times a week).

## Materials and Methods

### Study samples

A large scale longitudinal cohort study was set up in 2005 on middle-to-upper class urban Han Chinese who attended routine health checks at Centers for Health Management of Shandong Provincial Qianfoshan Hospital and of Shandong Provincial Hospital. A sub-cohort was selected from those free of MetS nor its components (obesity, dyslipidemia, hyperglycemia, and hypertension) at baseline. A total of 6,513 participants with at least three health checks in the six-year follow-up were included in our study. All individuals in the sub-cohort underwent a general health questionnaire, anthropometric, and laboratory test. The general health questionnaire covered smoking, alcohol intake, diet, sleeping quality and physical activity. The anthropometric tests included height, weight, and blood pressure, with both height and weight measured with light clothing without shoes. Body mass index (BMI) was calculated as weight/height^2^ (kg/m^2^) as an evidence of obesity. Blood pressure was measured on the right arm from a sitting position following a 5-min rest. Laboratory tests included total leukocyte/its subtypes, glucose, total cholesterol (TC), low-density lipoprotein (LDL), high-density lipoprotein (HDL), triglycerides (TG), uric acid(UA), gamma-glutamyl transpeptidase (GGT), serum albumin(ALB), serum globulins(GLO), blood urea nitrogen (BUN), serum creatinine (S-Cr), hemoglobin (Hb), hematokrit (HCT), mean corpuscular volume(MCV), mean corpuscular hemoglobin (MCH), red blood cell distribution width (RDW), platelet distribution width (PDW), mean platelet volume (MPV) and thrombocytocrit (PCT). This study was approved by the Ethics Committee of School of Public Health, Shandong University, and all participants gave informed written consent.

**Table 2:** Results after adjusting for potential confounding factors.

Quartiles	Estimate	Z	Pr>|Z|	RR	lower 95% Confidence Limits	upper 95% Confidence Limits
leukocyte						
Q4	0.9765	4.99	<0.0001	2.6551	1.8089	3.8970
Q3	0.4642	2.25	0.0243	1.5907	1.0622	2.3824
Q2	0.3166	1.49	0.1362	1.3725	0.905	2.0815
Q1	ref	ref	ref	ref	1	1
age	0.0209	3.30	0.0010	1.0211	1.0085	1.0339
gender	0.0376	0.15	0.8776	1.0383	0.6438	1.6743
time	0.2164	5.58	<0.0001	1.2416	1.1507	1.3398
GGT	0.0102	6.43	<0.0001	1.0103	1.0071	1.0134
TP	−0.0028	−0.04	0.9697	0.9972	0.8624	1.1530
ALB	−0.0168	−0.18	0.8534	0.9833	0.8232	1.1747
GLO	−0.0396	−0.43	0.6646	0.9612	0.8034	1.1498
A/G	−1.2350	−1.17	0.2430	0.2908	0.0366	2.3120
BUN	0.1773	4.10	<0.0001	1.194	1.0970	1.2995
TC	0.3734	5.54	<0.0001	1.4527	1.2728	1.6580
HB	−0.0244	−0.21	0.8320	0.9759	0.7793	1.2223
HCT	0.1672	0.43	0.6648	1.182	0.5547	2.5186
MCV	−0.1007	−6.00	<0.0001	0.9042	0.8749	0.9344
diet	0.0367	0.54	0.5892	1.0374	0.9080	1.1852
drinking	0.0566	1.26	0.2087	1.0582	0.9688	1.1559
smoking	0.0170	0.44	0.6584	1.0171	0.9433	1.0969

**Table 3:** Results after adjusting for other potential confounding factors.

Quartiles	Estimate	Z	Pr>|Z|	RR	lower 95% Confidence Limits	upper 95% Confidence Limits
lymphcyte						
Q4	0.5087	2.67	0.0076	1.6631	1.1447	2.4165
Q3	−0.1341	−0.65	0.5142	0.8745	0.5845	1.3084
Q2	−0.0655	−0.33	0.7428	0.9366	0.6332	1.3851
Q1	ref	ref	ref	ref	1	1
monocyte						
Q4	0.0115	0.05	0.9596	1.0116	0.6485	1.5779
Q3	0.2066	0.97	0.3298	1.2295	0.8116	1.8625
Q2	0.1120	0.54	0.5875	1.1185	0.7463	1.6762
Q1	ref	ref	ref	ref	1	1
neutrophils						
Q4	0.7416	3.84	0.0001	2.0993	1.4373	3.0658
Q3	0.1789	0.91	0.3644	1.1959	0.8125	1.7602
Q2	0.0975	0.5	0.6185	1.1024	0.7511	1.6179
Q1	ref	ref	ref	ref	1	1
age	0.0221	3.44	0.0006	1.0223	1.0095	1.0352
gender	0.0301	0.12	0.9025	1.0306	0.6372	1.6668
time	0.2079	5.21	<0.0010	1.2311	1.1384	1.3314
GGT	0.0105	6.33	<0.0010	1.0106	1.0073	1.0139
ALB	−0.0195	−0.21	0.8375	0.9807	0.8140	1.1815
GLO	−0.0464	−0.50	0.6200	0.9547	0.7946	1.1469
BUN	0.1788	4.12	<0.001	1.1958	1.0982	1.3020
TC	0.3706	5.45	<0.001	1.4486	1.2679	1.6550
Hb	−0.0273	−0.24	0.8133	0.9731	0.7755	1.2208
HCT	0.1733	0.45	0.6559	1.1892	0.5549	2.5482
MCV	−0.1012	−5.91	<0.001	0.9038	0.8739	0.9345
diet	0.0385	0.57	0.5706	1.0393	0.9097	1.1873
drinking	0.0616	1.34	0.1790	1.0635	0.9721	1.1636
smoking	0.0139	0.35	0.7267	1.0140	0.9382	1.0958

### Definition of Metabolic Syndrome

According to the criteria given by the Chinese Medical Association Diabetes Branch (CDS) designed for Chinese [28], MetS was defined as presence of three or more of the following four risk factors: 1) obesity or overweight, BMI≥25 0kg/m^2^; 2) hypertension, systolic/diastolic blood pressure ≥140/90 mmHg or previous diagnosis; 3) dyslipidemia, defined as fasting TG≥1.7 mmol/L(110 mg/dl), or fasting high-density(HDL)<0.9 mmol/L(35 mg/dl); 4) hyperglycemia, fasting blood-glucose (FPG)≥6.1 mmol/L(110 mg/dl) or 2 h Post-meal glucose (PG)≥7.8 mmol/L(140 mg/dl), or previous diagnosis.

### Statistical Analysis

#### Missing Data Imputation

To account for missing values, multiple imputation was performed. Since imputation method of choice depended on the pattern of missingness and the type of the imputed variables, without loss of generality the Markov chain Monte Carlo (MCMC) method was chosen according to MI Procedure of SAS 9.2[29]. After imputation, all variables had less than 10% missing observations, in particular, less than 2% for the total leukocyte and its subtypes, and 1% for other variables except diet, drinking, smoking, quality of sleep and physical activity.

#### Data analysis

Summary statistics were obtained for variables of interest at both baseline and follow-up. Simple GEE model was first used to select factors associated with MetS, and multiple GEE model was further adopted to detect the association between leukocyte/its subtypes counts and MetS/its components [30]–[33].

Variables which were significant at the significant level of 0.05 (α) in the simple GEE analysis entered the multiple GEE model to adjust the potential confounding. The GEE models used ‘Logit’ as the link function, adjusted for baseline age, and those *P*<0.05 were considered significant. All statistical analysis was performed by SAS 9.2.

## Results

Shown in Table 1 are the characteristics of the total leukocyte/its subtype, together with potential confounding factors at baseline and each follow-up year. This indicated that they were generally higher than baseline (P<0.05), though some did not reach statistical significance. A total of 255 cases of MetS occurred over the six-year follow-up, leading to a total incidence density of 11.45 per 1,000 person-years (255/22279 person-years), with 1.7, 9, 12.6,18.2 and 19.6 per 1,000 person-years of follow-up in the 1st, 2nd, 3rd, 4th and 5th year, respectively.

Before GEE analysis, the total leukocyte/its subtype were orderly discretized by *P*_25_, *P*_50_ *P*_75_ quartiles (see Table S1). Shown in Table S2 to S6 were the results of the simple GEE analysis. This suggesting that a) Age, gender, Leukocyte, Lymphocyte, Monocyte, Neutrophil, GGT, ALB, GLO, BUN, TC, Hb, HCT, MCH, diet, drinking and smoking were associated with MetS(see Table S2); b)All variables except basophil, PDW, MPV, PCT, quality of sleep and physical activity with obesity (see Table S3); c) All variables except basophil, PDW and PCT with dyslipidemia (see Table S4); d) All variables except eosnophils, basophil, PDW, MPV, PCT, drinking, quality of sleep and exercise with hyperglycemia (see Table S5); e) All variables except leukocyte, lymphocyte, monocyte, neutrophil, eosnophils, GGT, MCH, diet, drinking, smoking and quality of sleep with hypertension (see Table S6).

Shown in Table 2 were the association between the total leukocyte and MetS after adjusting the potential confounders in the multiple GEE analysis, such that it was associated with MetS at the top quartile (RR = 2.66, 95%CI = 1.81–3.90); p<0.0001), the third quartile (RR = 1.60, 95%CI = 1.06–2.38); p = 0.0243) levels using the Q1 as reference level. Although not significant at Q2, a trend of increase of RR was observed from Q2 to Q4. Shown in Table 3 was the association between the leukocyte subtypes and MetS after adjusting the potential confounders in the multiple GEE analysis, suggesting that similar trends can be found in monocytes, lymphocytes, and neutrophils compared with the total leukocyte, though some levels did not reach statistical significance.

The association of the total leukocyte/its subtypes with single MetS component (obesity, diabetes, hypertension and dyslipidemia) were further explored and shown in Tables S7, S8, S9, S10, S11, S12, S13. The total leukocyte, neutrophil, monocyte and eosinophil levels were strong and independent risk factors to obesity and an obvious increased trend of RR are revealed from Q2 to Q4 after adjusting the potential confounding factors (see Table S7 and Table S8). For hypertension, neither total leukocyte nor its subtypes were statistically significant (see Table S9). Nevertheless, the total leukocyte and neutrophil were strong and independent risk factors to dyslipidemia(see Table S10 and Table S11). Finally, for hyperglycemia, the total leukocyte and neutrophil were significant, though not at the second quartile (see Table S12 and Table S13).

## Discussion

This work mainly focus on detecting the association between the total leukocyte/ its subtypes and MetS/its components using the routine health-check based longitudinal cohort of urban Han Chinese population from middle to upper socioeconomic strata. The total incidence density was 11.45 per 1,000 person-years, with 1.7, 9, 12.6, 18.2 and 19.6 per 1,000 person-years of follow-up in the 1st, 2nd, 3rd, 4th and 5th year, respectively. Similar trend was reported in Japanese employee population [26].

We found that the total leukocyte was strongly associated with MetS and a dose-response existed (see Table 2). Furthermore, similar trends can be found in monocytes, lymphocytes, and neutrophils compared with the total leukocyte, though some levels did not reach statistical significance (see Table 3). In addition, similar findings were also reported by different study in different population, including a health check-up cohort in South China [25], Japanese employee population [26], and black-white population (preadolescents, adolescents, and adults)[16], These consistent conclusions indicated that the total leukocyte/its subtype may provide convenient and useful markers for further risk appraisal of MetS.

The associations of the total leukocyte/its subtype with single MetS component (obesity, diabetes, hypertension and dyslipidemia) were also identified in this paper. The total leukocyte, neutrophil, monocyte and eosinophil levels were strong and independent risk factors to obesity (see Table S7 and Table S8), total leukocyte and neutrophil to dyslipidemia(see Table S10 and Table S11)and hyperglycemia (see Table S12 and Table S13), while neither total leukocyte nor its subtypes to hypertension (see Table S9). Our finding confirmed the conclusions drawn from previous cross-section studies [18], [20], [34], [34,35,35]. Therefore, our results highlighted the association between the total leukocyte/its subtype and a variety of features of the MetS, indicating that they may be the earlier biomarkers for predicting cardiovascular disease than the components of MetS.

Although the mechanism of the relationship between leukocyte and metabolic syndrome remained unclear, several explanations have been offered: a) Insulin resistance played an important role in metabolic disturbances [36], [37], leading to higher gathering of inflammatory markers, including the total leukocyte and other inflammatory factors, such as CRP, IL-6, tumor necrosis factor-α (TNF-α), As a result, statistical association between the total leukocyte and MetS in population can be detected [38], [39]. b) Both obesity and dyslipidemia were the major precursors for development of MetS, and perivascular white adipose tissue can release proinflammatory cytokines [40], such as IL-8, leading to elevated leukocyte, especially the monocytes and granulocytes. In addition, TNF-α is shown to be constitutively expressed by adipose tissue, and this proinflammatory cytokines leads to elevated leukocyte [41]–[43]. Therefore, the total leukocyte/its subtype were associated with MetS. c) Hypertension, hyperlipidemia or hyperglycemia were the major precursors of MetS which can damage vascular endothelial. Furthermore, vascular endothelial cells can produce intracellular adhesion molecule-1, which caused the leukocyte to adhere to the vascular wall [18], [44], resulting production of new cytokines and chemokines, then cytokines were potential inducers of leukocyte differentiation [45]. In return, activated differentiated leukocyte can produce more cytokines [46]–[48]. Neutrophils can aggregate and release damaging substances, such as free radicals and proteolytic enzymes. Free radicals can lead to dysfunction of vascular endothelial cells, and a vicious circle was built. Thus the statistical association between leukocyte (especially neutrophils) and MetS was discovered.

A limitation of this study lie in the fact that use of routine health check-up population for middle-to-upper class urban Han Chinese can be subject to selection bias. Further investigation using general population is preferable. Due to the disadvantage of our routine health check-up database, we were unable to access the medical history of the participants. Owing to the absence of waist circumference measurement, the diagnostic criteria of MetS just based China Diabetes Federation, rather than international Standard criteria.

In conclusion, the total leukocyte/its subtype may provide convenient and useful markers for further risk appraisal of MetS, and they might be the earlier biomarkers for predicting cardiovascular disease than the components of MetS.

## Supporting Information

Table S1 **The quartiles of the total leukocyte/its subtypes.**
(DOCX)

Table S2 **The associated variables with MetS selected by the simple GEE model.**
(DOCX)

Table S3 **The associated variables with obesity selected by the simple GEE model.**
(DOC)

Table S4 **The associated variables with dyslipidemia selected by the simple GEE model.**
(DOC)

Table S5 **The associated variables with hyperglycemia selected by the simple GEE model.**
(DOC)

Table S6 **The associated variables with hypertension selected by the simple GEE model.**
(DOC)

Table S7 **Multiple GEE analysis of leukocyte and obesity after adjusting other potential confounding factors.**
(DOC)

Table S8 **Multiple GEE analysis of leukocyte subtypes and obesity after adjusting other potential confounding factors.**
(DOC)

Table S9 **Multiple GEE analysis of basophil and hypertension after adjusting other potential confounding factors.**
(DOC)

Table S10 **Multiple GEE analysis of leukocyte and dyslipidemia after adjusting other potential confounding factors.**
(DOCX)

Table S11 **Multiple GEE analysis of leukocyte subtypes and dyslipidemia after adjusting other potential confounding factors.**
(DOC)

Table S12 **Multiple GEE analysis of leukocyte and hyperglycemia after adjusting potential confounding factors.**
(DOC)

Table S13 **Multiple GEE analysis of leukocyte subtypes and hyperglycemia after adjusting other potential confounding factors.**
(DOCX)
