# Supplementary material for: Association between Leukocyte and Metabolic Syndrome in Urban Han Chinese: A Longitudinal Cohort Study
Source: PLoS One. 2012 Nov 27;7(11):e49875. doi: 10.1371/journal.pone.0049875 (PMC3507923; doi:10.1371/journal.pone.0049875)
Supplement: Table S1 — The quartiles of the total leukocyte/its subtypes. (DOCX) [file pone.0049875.s001.docx]

**TableS 1 The quartiles of the total leukocyte/its subtypes**

| **variables** | **Assignments** | | | | | | |
| --- | --- | --- | --- | --- | --- | --- | --- |
| WBC | qualified by their quartiles of P_25_, P_50_ and P_75_ every year; **Q1**: the UA level ≤ P_25_, **Q2**: P_25_ < the UA level < P_50_, **Q3**: P_50_ < the UA level < P_75_, **Q4**: the UA level ≥ P_75_ | | | | | | |
|  |  | 2005 | 2006 | 2007 | 2008 | 2009 | 2010 |
|  | P25 | 5.27 | 5.17 | 5.04 | 5.02 | 5.11 | 5.16 |
|  | P50 | 6.10 | 6.02 | 5.89 | 5.85 | 6.01 | 6.04 |
|  | P75 | 7.06 | 7.00 | 6.87 | 6.86 | 7.01 | 7.03 |
| [lymphocyte](app:ds:lymphocyte) |  |  |  |  |  |  |  |
|  |  | 2005 | 2006 | 2007 | 2008 | 2009 | 2010 |
|  | P25 | 1.69 | 1.53 | 1.48 | 1.52 | 1.55 | 1.80 |
|  | P50 | 2.01 | 1.82 | 1.79 | 1.85 | 1.91 | 2.15 |
|  | P75 | 2.42 | 2.15 | 2.17 | 2.52 | 2.38 | 2.55 |
| monocyte |  |  |  |  |  |  |  |
|  |  | 2005 | 2006 | 2007 | 2008 | 2009 | 2010 |
|  | P25 | 0.29 | 0.27 | 0.25 | 0.25 | 0.25 | 0.28 |
|  | P50 | 0.35 | 0.33 | 0.31 | 0.31 | 0.32 | 0.34 |
|  | P75 | 0.43 | 0.41 | 0.39 | 0.39 | 0.40 | 0.42 |
| [neutrophil](app:ds:neutrophil) |  |  |  |  |  |  |  |
|  |  | 2005 | 2006 | 2007 | 2008 | 2009 | 2010 |
|  | P25 | 2.88 | 3.02 | 2.93 | 2.87 | 2.89 | 2.65 |
|  | P50 | 3.47 | 3.65 | 3.54 | 3.45 | 3.52 | 3.29 |
|  | P75 | 4.25 | 4.38 | 4.32 | 4.20 | 4.28 | 4.03 |
| [eosnophils](app:ds:Eosnophils) |  |  |  |  |  |  |  |
|  |  | 2005 | 2006 | 2007 | 2008 | 2009 | 2010 |
|  | P25 | 0.07 | 0.06 | 0.06 | 0.06 | 0.06 | 0.06 |
|  | P50 | 0.11 | 0.10 | 0.10 | 0.10 | 0.10 | 0.10 |
|  | P75 | 0.18 | 0.17 | 0.18 | 0.17 | 0.18 | 0.17 |
| basophil |  |  |  |  |  |  |  |
|  |  | 2005 | 2006 | 2007 | 2008 | 2009 | 2010 |
|  | P25 | 0.01 | 0.01 | 0.01 | 0.01 | 0.01 | 0.02 |
|  | P50 | 0.02 | 0.02 | 0.02 | 0.01 | 0.01 | 0.02 |
|  | P75 | 0.03 | 0.03 | 0.03 | 0.02 | 0.02 | 0.03 |
