# Supplementary material for: Association between Leukocyte and Metabolic Syndrome in Urban Han Chinese: A Longitudinal Cohort Study
Source: PLoS One. 2012 Nov 27;7(11):e49875. doi: 10.1371/journal.pone.0049875 (PMC3507923; doi:10.1371/journal.pone.0049875)
Supplement: Table S2 — The associated variables with MetS selected by the simple GEE model. (DOCX) [file pone.0049875.s002.docx]

**Table S2 The associated variables with MetS selected by the simple GEE model**

| **Quartiles** | **Estimate** | **Error** | **Z** | **Pr>\|Z\|** | **RR** | **lower 95% Confidence Limits** | **upper 95% Confidence Limits** |
| --- | --- | --- | --- | --- | --- | --- | --- |
| Leukocyte | 0.2757 | 0.0309 | 8.92 | <0.0001 | 1.3175 | 1.0175 | 1.0396 |
| Lymphocyte | 0.6800 | 0.0896 | 7.59 | <0.0001 | 1.9739 | 1.0175 | 1.0396 |
| Monocyte | 2.0417 | 0.4283 | 4.77 | <0.0001 | 7.7037 | 1.0175 | 1.0396 |
| Neutrophil | 0.2561 | 0.0365 | 7.01 | <0.0001 | 1.2919 | 1.0175 | 1.0396 |
| eosnophil | 0.6820 | 0.4103 | 1.66 | 0.0964 | 1.9778 | 1.0175 | 1.0396 |
| Basophil | 4.9646 | 3.2180 | 1.54 | 0.1229 | 143.2512 | 1.0175 | 1.0396 |
| Age | 0.0280 | 0.0055 | 5.11 | <0.0001 | 1.0284 | 1.0175 | 1.0396 |
| Gender | -1.0846 | 0.1447 | -7.49 | <0.0001 | 0.3380 | 1.0175 | 1.0396 |
| GGT | 0.0134 | 0.0017 | 7.93 | <0.0001 | 1.0135 | 1.0175 | 1.0396 |
| ALB | -0.0726 | 0.0224 | -3.24 | 0.0012 | 0.9300 | 1.0175 | 1.0396 |
| GLO | 0.0792 | 0.0140 | 5.65 | <0.0001 | 1.0824 | 1.0175 | 1.0396 |
| BUN | 0.2562 | 0.0480 | 5.33 | <0.0001 | 1.2920 | 1.0175 | 1.0396 |
| SCr | 0.0132 | 0.0070 | 1.88 | 0.0606 | 1.0133 | 1.0175 | 1.0396 |
| TC | 0.5391 | 0.0636 | 8.48 | <0.0001 | 1.7145 | 1.0175 | 1.0396 |
| Hb | 0.0332 | 0.0083 | 3.99 | <0.0001 | 1.0338 | 1.0175 | 1.0396 |
| HCT | 0.1126 | 0.0185 | 6.10 | <0.0001 | 1.1192 | 1.0175 | 1.0396 |
| MCV | -0.0396 | 0.0138 | -2.87 | 0.0041 | 0.9612 | 1.0175 | 1.0396 |
| MCH | 0.0429 | 0.0426 | 1.01 | 0.3144 | 1.0438 | 1.0175 | 1.0396 |
| RDW | -0.0627 | 0.0787 | -0.80 | 0.4256 | 0.9392 | 1.0175 | 1.0396 |
| PDW | -0.004 | 0.0408 | -0.10 | 0.9226 | 0.9960 | 1.0175 | 1.0396 |
| MPV | -0.1096 | 0.0912 | -1.20 | 0.2295 | 0.8962 | 1.0175 | 1.0396 |
| PCT | -0.9755 | 1.0757 | -0.91 | 0.3645 | 0.3770 | 1.0175 | 1.0396 |
| diet | 0.1875 | 0.0720 | 2.61 | 0.0092 | 1.2062 | 1.0175 | 1.0396 |
| drinking | 0.1731 | 0.0434 | 3.99 | <0.0001 | 1.1890 | 1.0175 | 1.0396 |
| smoking | 0.1592 | 0.0424 | 3.76 | 0.0002 | 1.1726 | 1.0175 | 1.0396 |
| sleep | 0.1462 | 0.0785 | 1.86 | 0.0625 | 1.1574 | 1.0175 | 1.0396 |
| Physical activity | 0.1363 | 0.1429 | 0.95 | 0.3402 | 1.1460 | 1.0175 | 1.0396 |
