# Supplementary material for: Association between Leukocyte and Metabolic Syndrome in Urban Han Chinese: A Longitudinal Cohort Study
Source: PLoS One. 2012 Nov 27;7(11):e49875. doi: 10.1371/journal.pone.0049875 (PMC3507923; doi:10.1371/journal.pone.0049875)
Supplement: Table S3 — The associated variables with obesity selected by the simple GEE model. (DOC) [file pone.0049875.s003.doc]

**Table S3 The associated variables with obesity selected by the simple GEE model**

| **Variable** | **Estimate** | **Error** | **Z** | **Pr>|Z|** | **RR** | **Lower 95% confidence limit** | **Upper 95% confidence limit** |
| --- | --- | --- | --- | --- | --- | --- | --- |
| Leukocytye | 0.1752 | 0.0183 | 9.56 | <0.0001 | 1.1915 | 1.1495 | 1.2352 |
| Lymphocyte | 0.3395 | 0.048 | 7.08 | <0.0001 | 1.4042 | 1.2783 | 1.5426 |
| Monocyte | 1.4873 | 0.218 | 6.82 | <0.0001 | 4.4251 | 2.8864 | 6.7849 |
| Neutrophil | 0.1647 | 0.023 | 7.17 | <0.0001 | 1.1790 | 1.1272 | 1.2333 |
| Eosnophil | 0.5971 | 0.1923 | 3.11 | 0.0019 | 1.8168 | 1.2486 | 2.6485 |
| Basophil | 2.6734 | -1.4654 | 1.82 | 0.0681 | 14.4891 | 1.2198 | 256.0826 |
| age | 0.0081 | 0.0025 | 3.26 | 0.0011 | 1.0081 | 1.0032 | 1.0131 |
| gender | -0.7729 | 0.0598 | -12.92 | <0.0001 | 0.4617 | 0.4106 | 0.5191 |
| GGT | 0.0100 | 0.0014 | 7.31 | <0.0001 | 1.0101 | 1.0073 | 1.0127 |
| ALB | -0.0698 | -0.0109 | -6.41 | <0.0001 | 0.9326 | 1.0954 | 0.9527 |
| GLO | 0.0440 | 0.0070 | 6.3 | <0.0001 | 1.0450 | 1.0308 | 1.0594 |
| BUN | 0.0868 | 0.0251 | 3.45 | 0.0006 | 1.0907 | 1.0382 | 1.1458 |
| SCr | 0.0105 | 0.0036 | 2.93 | 0.0034 | 1.0106 | 1.0035 | 1.0178 |
| TC | 0.2435 | 0.0320 | 7.62 | <0.0001 | 1.2757 | 1.1982 | 1.3581 |
| Hb | 0.0176 | 0.0022 | 7.94 | <0.0001 | 1.0178 | 1.0133 | 1.0221 |
| HCT | 0.0429 | 0.0083 | 5.19 | <0.0001 | 1.0438 | 1.0271 | 1.0609 |
| MCV | -0.0157 | 0.0062 | -2.54 | 0.0111 | 0.9844 | 0.9726 | 0.9964 |
| MCH | 0.0412 | 0.0167 | 2.46 | 0.0139 | 1.0421 | 1.0084 | 1.0768 |
| RDW | 0.0189 | 0.0026 | 7.22 | <0.0001 | 1.0191 | 1.0139 | 1.0244 |
| PDW | -0.0187 | 0.0199 | -0.94 | 0.3461 | 0.9815 | 0.9439 | 1.0204 |
| MPV | -0.0663 | 0.0412 | -1.61 | 0.1077 | 0.9359 | 0.8633 | 1.0146 |
| PCT | 0.1207 | -0.1422 | 0.85 | 0.3960 | 1.1283 | 1.1712 | 1.4909 |
| diet | 0.2223 | 0.0348 | 6.38 | <0.0001 | 1.2489 | 1.1665 | 1.3371 |
| drinking | 0.1250 | 0.0231 | 5.42 | <0.0001 | 1.1331 | 1.0831 | 1.1855 |
| smoking | 0.0824 | 0.0258 | 3.20 | 0.0014 | 1.0859 | 1.0323 | 1.1421 |
| sleep | -0.0393 | -0.0416 | -0.94 | 0.3447 | 0.9615 | 1.1286 | 1.0432 |
| Physical Activity | 0.0042 | 0.0753 | 0.06 | 0.9556 | 1.0042 | 0.8664 | 1.1639 |
