# Supplementary material for: Association between Leukocyte and Metabolic Syndrome in Urban Han Chinese: A Longitudinal Cohort Study
Source: PLoS One. 2012 Nov 27;7(11):e49875. doi: 10.1371/journal.pone.0049875 (PMC3507923; doi:10.1371/journal.pone.0049875)
Supplement: Table S4 — The associated variables with dyslipidemia selected by the simple GEE model. (DOC) [file pone.0049875.s004.doc]

**Table S4 The associated variables with dyslipidemia selected by the simple GEE model**

| **Variable** | **Estimate** | **Error** | **Z** | **Pr>|Z|** | **RR** | **Lower 95% confidence limit** | **Upper 95% confidence limit** |
| --- | --- | --- | --- | --- | --- | --- | --- |
| Leukocyte | 0.1831 | 0.0118 | 15.57 | <0.0001 | 1.2009 | 1.1736 | 1.2290 |
| lymphocyte | 0.3564 | 0.0310 | 11.51 | <0.0001 | 1.4282 | 1.3441 | 1.5176 |
| Monocyte | 1.3257 | 0.1473 | 9.00 | <0.0001 | 3.7648 | 2.8210 | 4.2207 |
| Neutrophil | 0.1769 | 0.0149 | 11.90 | <0.0001 | 1.1935 | 1.1593 | 1.2288 |
| Eosnophil | 1.0727 | 0.1288 | 8.33 | <0.0001 | 2.9233 | 2.2710 | 3.7633 |
| Basophil | -1.0530 | 1.0730 | -0.98 | 0.3264 | 0.3489 | 0.0426 | 2.8579 |
| age | -0.0070 | 0.0016 | -4.27 | <0.0001 | 0.993 | 0.9899 | 0.9962 |
| Gender | -0.5730 | 0.0370 | -15.50 | <0.0001 | 0.5638 | 0.5244 | 0.6062 |
| GGT | 0.0205 | 0.0016 | 12.61 | <0.0001 | 1.0207 | 1.0175 | 1.0240 |
| ALB | 0.0271 | 0.0073 | 3.73 | 0.0002 | 1.0275 | 1.0129 | 1.0422 |
| GLO | 0.0153 | 0.0045 | 3.43 | 0.0006 | 1.0154 | 1.0066 | 1.0244 |
| BUN | 0.0721 | 0.0151 | 4.77 | <0.0001 | 1.0748 | 1.0434 | 1.1071 |
| SCr | 0.0165 | 0.0021 | 7.71 | <0.0001 | 1.0166 | 1.0124 | 1.0210 |
| TC | 0.1777 | 0.0219 | 8.12 | <0.0001 | 1.1945 | 1.1443 | 1.2469 |
| Hb | 0.0217 | 0.0014 | 15.06 | <0.0001 | 1.0219 | 1.0191 | 1.0249 |
| HCT | 0.0725 | 0.0049 | 14.75 | <0.0001 | 1.0752 | 1.0649 | 1.0856 |
| MCV | -0.0168 | 0.0036 | -4.65 | <0.0001 | 0.9833 | 0.9764 | 1.0097 |
| MCH | 0.0187 | 0.0088 | 2.11 | 0.0346 | 1.0189 | 1.0014 | 1.0367 |
| RDW | 0.0177 | 0.0016 | 11.15 | <0.0001 | 1.0179 | 1.0147 | 1.0211 |
| PDW | 0.0042 | 0.0109 | 0.38 | 0.7023 | 1.0042 | 0.9828 | 1.0259 |
| MPV | -0.0930 | 0.0233 | -3.98 | <0.0001 | 0.9112 | 0.8705 | 0.9539 |
| PCT | 0.1405 | 0.1104 | 1.27 | 0.2032 | 1.1508 | 0.9269 | 1.4290 |
| diet | 0.2095 | 0.0211 | 9.95 | <0.0001 | 1.2331 | 1.1833 | 1.2851 |
| drinking | 0.1842 | 0.0119 | 15.52 | <0.0001 | 1.2023 | 1.1747 | 1.2306 |
| smoking | 0.1578 | 0.0112 | 14.13 | <0.0001 | 1.1709 | 1.1456 | 1.1969 |
| sleep | 0.1083 | 0.0221 | 4.90 | <0.0001 | 1.1144 | 1.0672 | 1.1637 |
| Physical activity | -0.2283 | 0.0420 | -5.44 | <0.0001 | 0.7959 | 0.7330 | 0.8642 |
