# Supplementary material for: Association between Leukocyte and Metabolic Syndrome in Urban Han Chinese: A Longitudinal Cohort Study
Source: PLoS One. 2012 Nov 27;7(11):e49875. doi: 10.1371/journal.pone.0049875 (PMC3507923; doi:10.1371/journal.pone.0049875)
Supplement: Table S5 — The associated variables with hyperglycemia selected by the simple GEE model. (DOC) [file pone.0049875.s005.doc]

**Table S5 The associated variables with hyperglycemia selected by the simple GEE model**

| **Variable** | **Estimate** | **Error** | **Z** | **Pr>|Z|** | **RR** | **Lower 95% confidence limit** | **Upper 95% confidence limit** |
| --- | --- | --- | --- | --- | --- | --- | --- |
| Leukocyte | 0.1621 | 0.0232 | 7.00 | <0.0001 | 1.176 | 1.1238 | 1.2306 |
| lymphocyte | 0.3792 | 0.0612 | 6.20 | <0.0001 | 1.4611 | 1.2960 | 1.6472 |
| monocyte | 1.2963 | 0.3117 | 4.16 | <0.0001 | 3.6557 | 1.9846 | 6.7342 |
| neutrophil | 0.1479 | 0.0290 | 5.10 | <0.0001 | 1.1594 | 1.0953 | 1.2273 |
| eosnophil | 0.1056 | 0.2802 | 0.38 | 0.7064 | 1.1114 | 0.6417 | 1.9248 |
| basophil | 2.4937 | 2.0680 | 1.21 | 0.2279 | 12.106 | 0.2103 | 697.0102 |
| age | 0.0126 | 0.0037 | 3.40 | 0.0007 | 1.0127 | 1.0053 | 1.0201 |
| gender | -0.5378 | 0.0791 | -6.80 | <0.0001 | 0.5840 | 0.5001 | 0.6820 |
| GGT | 0.0093 | 0.0013 | 7.12 | <0.0001 | 1.0093 | 1.0067 | 1.0119 |
| ALB | -0.0717 | 0.0141 | -5.08 | <0.0001 | 0.9308 | 0.9054 | 0.9570 |
| GLO | 0.0767 | 0.0083 | 9.23 | <0.0001 | 1.0797 | 1.0623 | 1.0975 |
| BUN | 0.1633 | 0.0307 | 5.32 | <0.0001 | 1.1774 | 1.1086 | 1.2503 |
| SCr | 0.0091 | 0.0036 | 2.51 | 0.0121 | 1.0091 | 1.0020 | 1.0163 |
| TC | 0.4314 | 0.0406 | 10.62 | <0.0001 | 1.5394 | 1.4216 | 1.6670 |
| Hb | 0.0132 | 0.0028 | 4.71 | <0.0001 | 1.0133 | 1.0077 | 1.0189 |
| HCT | 0.0262 | 0.0105 | 2.50 | 0.0126 | 1.0265 | 1.0056 | 1.0479 |
| MCV | -0.0190 | 0.0089 | -2.13 | 0.0332 | 0.9812 | 0.9641 | 0.9985 |
| MCH | 0.0547 | 0.0255 | 2.15 | 0.0318 | 1.0562 | 1.0048 | 1.1104 |
| RDW | 0.0273 | 0.0038 | 7.23 | <0.0001 | 1.0277 | 1.0201 | 1.0353 |
| PDW | -0.0381 | 0.0246 | -1.55 | 0.1205 | 0.9626 | 0.9173 | 1.0101 |
| MPV | -0.0968 | 0.0514 | -1.88 | 0.0597 | 0.9077 | 0.8208 | 1.0039 |
| PCT | -0.0665 | 0.1855 | -0.36 | 0.7199 | 0.9357 | 0.6505 | 1.3458 |
| diet | 0.1935 | 0.0423 | 4.57 | <0.0001 | 1.2135 | 1.1168 | 1.3185 |
| drinking | 0.0229 | 0.0292 | 0.78 | 0.4328 | 1.0232 | 0.9663 | 1.0835 |
| smoking | 0.0861 | 0.0248 | 3.47 | 0.0005 | 1.0899 | 1.0382 | 1.1442 |
| sleep | 0.0287 | 0.0440 | 0.65 | 0.5142 | 1.0291 | 0.9441 | 1.1218 |
| Physical activity | -0.0383 | 0.0865 | -0.44 | 0.6582 | 0.9624 | 0.8124 | 1.1403 |
