# Supplementary material for: Association between Leukocyte and Metabolic Syndrome in Urban Han Chinese: A Longitudinal Cohort Study
Source: PLoS One. 2012 Nov 27;7(11):e49875. doi: 10.1371/journal.pone.0049875 (PMC3507923; doi:10.1371/journal.pone.0049875)
Supplement: Table S6 — The associated variables with hypertension selected by the simple GEE model. (DOC) [file pone.0049875.s006.doc]

**Table S6 The associated variables with hypertension selected by the simple GEE model**

| **Variable** | **Estimate** | **Error** | **Z** | **Pr>|Z|** | **RR** | **Lower 95% confidence limit** | **Upper 95% confidence limit** |
| --- | --- | --- | --- | --- | --- | --- | --- |
| Leukocyte | 0.0170 | 0.0115 | 1.48 | 0.1385 | 1.0171 | 0.9945 | 1.0403 |
| lymphocyte | 0.0255 | 0.0281 | 0.91 | 0.3641 | 1.0258 | 0.9709 | 1.0838 |
| Monocyte | 0.2147 | 0.1308 | 1.64 | 0.1006 | 1.2395 | 0.9593 | 1.6016 |
| Neutrophil | 0.0197 | 0.0131 | 1.51 | 0.1312 | 1.0199 | 0.9941 | 1.0463 |
| Eosnophil | -1.644 | 1.3281 | -1.24 | 0.2158 | 0.1932 | 0.0143 | 2.6091 |
| Basophil | 1.9805 | 0.7217 | 2.74 | 0.0061 | 7.2464 | 1.7610 | 29.8147 |
| age | 0.0587 | 0.0026 | 22.93 | <0.0001 | 1.0605 | 1.0552 | 1.0658 |
| Gender | -0.8007 | 0.0802 | -9.99 | <0.0001 | 0.4490 | 0.3837 | 0.5254 |
| GGT | 0.0178 | 0.0120 | 1.49 | 0.1371 | 1.0180 | 0.9943 | 1.0422 |
| ALB | 0.0288 | 0.0076 | 3.78 | 0.0002 | 1.0292 | 1.0140 | 1.0447 |
| GLO | 0.0611 | 0.0122 | 4.99 | <0.0001 | 1.0630 | 1.0378 | 1.0887 |
| TC | 0.3967 | 0.0268 | 14.82 | <0.0001 | 1.4869 | 1.4109 | 1.5669 |
| Hb | 0.0433 | 0.0157 | 2.76 | 0.0058 | 1.0443 | 1.0127 | 1.0769 |
| HCT | 0.0336 | 0.0084 | 4.01 | <0.0001 | 1.0342 | 1.0172 | 1.0513 |
| MCV | -0.0131 | 0.0043 | -3.09 | 0.0020 | 0.9870 | 0.9787 | 0.9952 |
| MCH | -0.0809 | 0.0626 | -1.29 | 0.1966 | 0.9223 | 0.8157 | 1.0428 |
| PDW | 0.0304 | 0.0108 | 2.82 | 0.0048 | 1.0309 | 1.0093 | 1.0528 |
| MPV | 0.0375 | 0.0173 | 2.16 | 0.0307 | 1.0382 | 1.0035 | 1.0741 |
| PCT | -1.1367 | 0.1877 | -6.06 | <0.0001 | 0.3209 | 0.2221 | 0.4635 |
| diet | -0.0142 | 0.0201 | -0.71 | 0.4798 | 0.9859 | 0.9478 | 1.0255 |
| drinking | -0.1933 | 0.1468 | -1.32 | 0.1879 | 0.8242 | 0.6181 | 1.0990 |
| smoking | 0.0303 | 0.0214 | 1.41 | 0.1579 | 1.0308 | 0.9884 | 1.0750 |
| sleep | -0.2934 | 0.1888 | -1.55 | 0.1202 | 0.7457 | 0.5151 | 1.0797 |
| Physical activity | -0.2031 | 0.0499 | -4.07 | <0.0001 | 0.8162 | 0.7402 | 0.9000 |
