# Supplementary material for: Association between Leukocyte and Metabolic Syndrome in Urban Han Chinese: A Longitudinal Cohort Study
Source: PLoS One. 2012 Nov 27;7(11):e49875. doi: 10.1371/journal.pone.0049875 (PMC3507923; doi:10.1371/journal.pone.0049875)
Supplement: Table S7 — Multiple GEE analysis of leukocyte and obesity after adjusting other potential confounding factors. (DOC) [file pone.0049875.s007.doc]

**Table S7 Multiple GEE analysis of leukocyte and obesity after** adjusting other potential confounding factors

| **Variable** | **Estimate** | **Error** | **Z** | **Pr>|Z|** | **RR** | **Lower 95% confidence limit** | **Upper 95% confidence limits** |
| --- | --- | --- | --- | --- | --- | --- | --- |
| **Leukocyte** | |  |  |  |  |  |  |
| Q4 | 0.6552 | 0.0741 | 8.84 | <0.0001 | 1.9255 | 1.6651 | 6.8588 |
| Q3 | 0.4217 | 0.0742 | 5.69 | <0.0001 | 1.5246 | 1.3184 | 4.5931 |
| Q2 | 0.2653 | 0.0757 | 3.51 | 0.0005 | 1.3038 | 1.1241 | 3.6833 |
| Q1 | ref | ref | ref | ref | ref | 1 | 1 |
| age | -0.0006 | 0.0024 | -0.26 | 0.7968 | 0.9994 | 0.9947 | 2.7167 |
| gender | -0.0601 | 0.0908 | -0.66 | 0.5083 | 0.9417 | 0.7881 | 2.5643 |
| time | 0.3087 | 0.0160 | 19.28 | <0.0001 | 1.3617 | 1.3196 | 3.9026 |
| GGT | 0.0096 | 0.0012 | 7.99 | <0.0001 | 1.0096 | 1.0072 | 2.7446 |
| ALB | -0.0666 | 0.0171 | -3.91 | <0.0001 | 0.9356 | 0.9048 | 2.5487 |
| GLO | -0.0434 | 0.0238 | -1.82 | 0.0683 | 0.9575 | 0.9138 | 2.6052 |
| BUN | 0.0404 | 0.0232 | 1.74 | 0.0817 | 1.0412 | 0.9949 | 2.8327 |
| SCr | 0.0038 | 0.0020 | 1.97 | 0.0493 | 1.0038 | 1 | 2.7287 |
| TC | 0.1216 | 0.0271 | 4.49 | <0.0001 | 1.1293 | 1.0709 | 3.0935 |
| Hb | 0.0165 | 0.0102 | 1.62 | 0.1055 | 1.0166 | 0.9965 | 2.7639 |
| HCT | 0.0020 | 0.0360 | 0.06 | 0.9553 | 1.0020 | 0.9338 | 2.7237 |
| MCV | -0.079 | 0.0851 | -0.93 | 0.3532 | 0.9240 | 0.7822 | 2.5194 |
| MCH | 0.1307 | 0.2574 | 0.51 | 0.6117 | 1.1396 | 0.6881 | 3.1256 |
| diet | 0.1557 | 0.0271 | 5.74 | <0.0001 | 1.1685 | 1.1079 | 3.2171 |
| drinking | 0.0673 | 0.0191 | 3.53 | 0.0004 | 1.0696 | 1.0305 | 2.9143 |
| smoking | -0.0397 | 0.0175 | -2.27 | 0.0231 | 0.9611 | 0.9288 | 2.6145 |
