# Supplementary material for: Association between Leukocyte and Metabolic Syndrome in Urban Han Chinese: A Longitudinal Cohort Study
Source: PLoS One. 2012 Nov 27;7(11):e49875. doi: 10.1371/journal.pone.0049875 (PMC3507923; doi:10.1371/journal.pone.0049875)
Supplement: Table S8 — Multiple GEE analysis of leukocyte subtypes and obesity after adjusting other potential confounding factors. (DOC) [file pone.0049875.s008.doc]

**Table S8 Multiple GEE analysis of leukocyte subtypes and obesity** after adjusting other potential confounding factors

| **Variable** | **Estimate** | **Error** | **Z** | **Pr>|Z|** | **RR** | **Lower 95% confidence limit** | **Upper 95% confidence limit** |
| --- | --- | --- | --- | --- | --- | --- | --- |
| **lymphocyte** | |  |  |  |  |  |  |
| Q4 | 0.1334 | 0.0756 | 1.76 | 0.0776 | 1.1427 | 0.9853 | 1.3252 |
| Q3 | -0.0010 | 0.0742 | -0.01 | 0.9893 | 0.9990 | 0.8637 | 1.1555 |
| Q2 | -0.0665 | 0.0743 | -0.89 | 0.3709 | 0.9357 | 0.809 | 1.0823 |
| Q1 | ref | ref | ref | ref | ref | 1 | 1 |
| **monocyte** | |  |  |  |  |  |  |
| Q4 | 0.0585 | 0.0836 | 0.7 | 0.4846 | 1.0602 | 0.8999 | 1.2491 |
| Q3 | 0.1710 | 0.0777 | 2.2 | 0.0277 | 1.1865 | 1.0189 | 1.3817 |
| Q2 | -0.0071 | 0.0751 | -0.09 | 0.9248 | 0.9929 | 0.8571 | 1.1504 |
| Q1 | ref | ref | ref | ref | ref | 1 | 1 |
| **neutrophil** | |  |  |  |  |  |  |
| Q4 | 0.5869 | 0.0764 | 7.68 | <0.0001 | 1.7984 | 1.5482 | 2.089 |
| Q3 | 0.4535 | 0.0737 | 6.16 | <0.0001 | 1.5738 | 1.3622 | 1.8181 |
| Q2 | 0.2729 | 0.0746 | 3.66 | 0.0003 | 1.3138 | 1.1351 | 1.5204 |
| Q1 | ref | ref | ref | ref | ref | 1 | 1 |
| **eosnophil** |  |  |  |  |  |  |  |
| Q4 | 0.1554 | 0.0713 | 2.18 | 0.0293 | 1.1681 | 1.0157 | 1.3433 |
| Q3 | 0.2319 | 0.0705 | 3.29 | 0.0010 | 1.2610 | 1.0983 | 1.4479 |
| Q2 | 0.1350 | 0.0711 | 1.90 | 0.0577 | 1.1445 | 0.9956 | 1.3156 |
| Q1 | ref | ref | ref | ref | ref | 1 | 1 |
| age | -0.0010 | 0.0024 | -0.42 | 0.6761 | 0.9990 | 0.9943 | 1.0037 |
| gender | -0.0577 | 0.0910 | -0.63 | 0.5261 | 0.9439 | 0.7897 | 1.1283 |
| time | 0.3138 | 0.0163 | 19.24 | <0.0001 | 1.3686 | 1.3256 | 1.4131 |
| GGT | 0.0096 | 0.0012 | 8.04 | <0.0001 | 1.0096 | 1.0073 | 1.0120 |
| TP | -0.0656 | 0.0170 | -3.85 | 0.0001 | 0.9365 | 0.9057 | 0.9683 |
| ALB | -0.0431 | 0.0237 | -1.82 | 0.0693 | 0.9578 | 0.9144 | 1.0034 |
| GLO | 0.0400 | 0.0233 | 1.72 | 0.0858 | 1.0408 | 0.9944 | 1.0894 |
| BUN | 0.1223 | 0.0271 | 4.51 | <0.0001 | 1.1301 | 1.0717 | 1.1918 |
| TC | 0.0156 | 0.0108 | 1.45 | 0.1474 | 1.0157 | 0.9945 | 1.0375 |
| HB | 0.0044 | 0.0379 | 0.11 | 0.9085 | 1.0044 | 0.9324 | 1.0819 |
| HCT | -0.0755 | 0.0857 | -0.88 | 0.3783 | 0.9273 | 0.7838 | 1.0969 |
| MCV | 0.1238 | 0.2594 | 0.48 | 0.6331 | 1.1318 | 0.6807 | 1.8819 |
| diet | 0.1585 | 0.0272 | 5.83 | <0.0001 | 1.1718 | 1.1110 | 1.2359 |
| drinking | 0.0677 | 0.0191 | 3.55 | 0.0004 | 1.0700 | 1.0308 | 1.1107 |
| smoking | -0.0435 | 0.0177 | -2.45 | 0.0143 | 0.9574 | 0.9247 | 0.9913 |
