# Supplementary material for: Association between Leukocyte and Metabolic Syndrome in Urban Han Chinese: A Longitudinal Cohort Study
Source: PLoS One. 2012 Nov 27;7(11):e49875. doi: 10.1371/journal.pone.0049875 (PMC3507923; doi:10.1371/journal.pone.0049875)
Supplement: Table S9 — Multiple GEE analysis of basophil and hypertension after adjusting other potential confounding factors. (DOC) [file pone.0049875.s009.doc]

**Table S9 Multiple GEE analysis of basophil and hypertension after** adjusting other potential confounding factors

| **Quartiles** | **Estimate** | **Error** | **Z** | **Pr>|Z|** | **RR** | **Lower 95% confidence limits** | **Upper 95% confidence limits** |
| --- | --- | --- | --- | --- | --- | --- | --- |
| **basophil** |  |  |  |  |  |  |  |
| Q4 | -0.0693 | 0.1123 | -0.62 | 0.5370 | 0.9330 | 0.7488 | 1.1626 |
| Q3 | -0.0676 | 0.1129 | -0.60 | 0.5492 | 0.9346 | 0.7490 | 1.1661 |
| Q2 | -0.0324 | 0.1089 | -0.30 | 0.766 | 0.9681 | 0.7820 | 1.1984 |
| Q1 | ref | ref | ref | ref | ref | 1 | 1 |
| age | 0.0600 | 0.0029 | 20.50 | <0.0001 | 1.0618 | 1.0557 | 1.0679 |
| gender | -0.0894 | 0.0969 | -0.92 | 0.3562 | 0.9145 | 0.7562 | 1.1057 |
| time | 0.3680 | 0.0212 | 17.4 | <0.0001 | 1.4448 | 1.3862 | 1.5061 |
| ALB | 0.4184 | 4.4171 | 0.09 | 0.9245 | 1.5195 | 0.0003 | 8.70E+03 |
| GLO | 0.4377 | 4.4161 | 0.10 | 0.9211 | 1.5491 | 0.0003 | 8.90E+03 |
| TC | 0.2495 | 0.0340 | 7.34 | <0.0001 | 1.2834 | 1.2007 | 1.3719 |
| Hb | 0.0489 | 0.0071 | 6.84 | <0.0001 | 1.0501 | 1.0355 | 1.0649 |
| HCT | -0.0973 | 0.0263 | -3.70 | 0.0002 | 0.9073 | 0.8617 | 0.9552 |
| MCV | -0.0282 | 0.0088 | -3.20 | 0.0014 | 0.9722 | 0.9556 | 0.9892 |
| RDW | 0.0122 | 0.0418 | 0.29 | 0.771 | 1.0123 | 0.9326 | 1.0988 |
| PDW | 0.0298 | 0.0583 | 0.51 | 0.6096 | 1.0302 | 0.9190 | 1.1550 |
| MPV | -0.3691 | 0.2797 | -1.32 | 0.187 | 0.6914 | 0.3996 | 1.1961 |
| PCT | 0.1476 | 0.0572 | 2.58 | 0.0099 | 1.159 | 1.0361 | 1.2965 |
| Physical activity | 0.1041 | 0.0658 | 1.58 | 0.1135 | 1.1097 | 0.9755 | 1.2624 |
