# Supplementary material for: Association between Leukocyte and Metabolic Syndrome in Urban Han Chinese: A Longitudinal Cohort Study
Source: PLoS One. 2012 Nov 27;7(11):e49875. doi: 10.1371/journal.pone.0049875 (PMC3507923; doi:10.1371/journal.pone.0049875)
Supplement: Table S10 — Multiple GEE analysis of leukocyte and dyslipidemia after adjusting other potential confounding factors. (DOCX) [file pone.0049875.s010.docx]

**Table S10 Multiple GEE analysis of leukocyte and dyslipidemia after adjusting other potential confounding factors**

| **Variable** | **Estimate** | **Error** | **Z** | **Pr>\|Z\|** | **RR** | **Lower 95% confidence limits** | **Upper 95% confidence limits** |
| --- | --- | --- | --- | --- | --- | --- | --- |
| **leukocyte** | |  |  |  |  |  |  |
| Q4 | 0.5364 | 0.2896 | 1.85 | 0.0640 | 1.71 | 0.97 | 3.02 |
| Q3 | 0.4913 | 0.2653 | 1.85 | 0.0641 | 1.63 | 0.97 | 2.75 |
| Q2 | 0.5218 | 0.2522 | 2.07 | 0.0386 | 1.69 | 1.03 | 2.76 |
| Q1 | ref | ref | ref | ref | ref | 1 | 1 |
| gender | 0.417 | 0.3461 | 1.2 | 0.2283 | 1.52 | 0.77 | 2.99 |
| time | 0.3201 | 0.0668 | 4.79 | <0.0001 | 1.38 | 1.21 | 1.57 |
| GGT | 0.0185 | 0.0039 | 4.79 | <0.0001 | 1.02 | 1.01 | 1.03 |
| GLO | 14.2871 | 0.1600 | 89.31 | <0.0001 | 1.60E+06 | 1.20E+06 | 2.20E+06 |
| BUN | -0.0666 | 0.0818 | -0.81 | 0.4155 | 0.94 | 0.8 | 1.1 |
| SCr | 0.015 | 0.0095 | 1.58 | 0.1152 | 1.02 | 1 | 1.03 |
| TC | 0.2542 | 0.0886 | 2.87 | 0.0041 | 1.29 | 1.08 | 1.53 |
| HB | 0.2779 | 0.1486 | 1.87 | 0.0614 | 1.32 | 0.99 | 1.77 |
| HCT | -0.8937 | 0.5009 | -1.78 | 0.0744 | 0.41 | 0.15 | 1.09 |
| MCV | 0.5313 | 0.3834 | 1.39 | 0.1658 | 1.7 | 0.8 | 3.61 |
| MCH | -1.5326 | 1.1377 | -1.35 | 0.1780 | 0.22 | 0.02 | 2.01 |
| PDW | -0.935 | 0.7720 | -1.21 | 0.2258 | 0.39 | 0.09 | 1.78 |
| PCT | 0.1165 | 0.0968 | 1.20 | 0.2290 | 1.12 | 0.93 | 1.36 |
| diet | 0.2516 | 0.1015 | 2.48 | 0.0132 | 1.29 | 1.05 | 1.57 |
| drinking | 0.1516 | 0.0565 | 2.68 | 0.0073 | 1.16 | 1.04 | 1.3 |
| smoking | 0.0606 | 0.0554 | 1.09 | 0.2738 | 1.06 | 0.95 | 1.18 |
| sleep | 0.0752 | 0.1192 | 0.63 | 0.5284 | 1.08 | 0.85 | 1.36 |
| Physical activity | -0.3598 | 0.1786 | -2.01 | 0.0439 | 0.70 | 0.49 | 0.99 |
