# Supplementary material for: Association between Leukocyte and Metabolic Syndrome in Urban Han Chinese: A Longitudinal Cohort Study
Source: PLoS One. 2012 Nov 27;7(11):e49875. doi: 10.1371/journal.pone.0049875 (PMC3507923; doi:10.1371/journal.pone.0049875)
Supplement: Table S11 — Multiple GEE analysis of leukocyte subtypes and dyslipidemia after adjusting other potential confounding factors. (DOC) [file pone.0049875.s011.doc]

**Table S11 Multiple GEE analysis of leukocyte subtypes and dyslipidemia after adjusting other potential confounding factors**

| **Variable** | **Estimate** | **Error** | **Z** | **Pr>|Z|** | **RR** | **Lower 95% confidence limits** | **Upper 95% confidence limits** |
| --- | --- | --- | --- | --- | --- | --- | --- |
| **lymphcyte** | |  |  |  |  |  |  |
| Q4 | 0.1027 | 0.2826 | 0.36 | 0.7164 | 1.11 | 0.64 | 1.93 |
| Q3 | 0.2027 | 0.2644 | 0.77 | 0.4431 | 1.22 | 0.73 | 2.06 |
| Q2 | 0.0625 | 0.2653 | 0.24 | 0.8139 | 1.06 | 0.63 | 1.79 |
| Q1 | ref | ref | ref | ref | ref | 1 | 1 |
| **monocyte** | |  |  |  |  |  |  |
| Q4 | -0.025 | 0.3265 | -0.08 | 0.9389 | 0.98 | 0.51 | 1.85 |
| Q3 | 0.3788 | 0.2983 | 1.27 | 0.2041 | 1.46 | 0.81 | 2.62 |
| Q2 | -0.0875 | 0.2786 | -0.31 | 0.7535 | 0.92 | 0.53 | 1.58 |
| Q1 | ref | ref | ref | ref | ref | 1 | 1 |
| **neutrophil** | |  |  |  |  |  |  |
| Q4 | 0.0617 | 0.2847 | 0.22 | 0.0285 | 1.16 | 1.01 | 1.86 |
| Q3 | 0.1194 | 0.2463 | 0.48 | 0.6278 | 1.13 | 0.70 | 1.83 |
| Q2 | 0.0449 | 0.2392 | 0.19 | 0.8510 | 1.05 | 0.65 | 1.67 |
| Q1 | ref | ref | ref | ref | ref | 1 | 1 |
| **eosnophil**  Q4 |  |  |  |  |  |  |  |
|  | 0.2051 | 0.2513 | 0.82 | 0.4145 | 1.23 | 0.75 | 2.01 |
| Q3 | 0.2249 | 0.2503 | 0.90 | 0.3688 | 1.25 | 0.77 | 2.05 |
| Q2 | 0.0356 | 0.2669 | 0.13 | 0.8938 | 1.04 | 0.61 | 1.75 |
| Q1 | ref | ref | ref | ref | ref | 1 | 1 |
| gender | 0.4389 | 0.345 | 1.27 | 0.2033 | 1.55 | 0.79 | 3.05 |
| time | 0.3308 | 0.0683 | 4.85 | <.0001 | 1.39 | 1.22 | 1.59 |
| GGT | 0.0181 | 0.0038 | 4.71 | <.0001 | 1.02 | 1.01 | 1.03 |
| ALB | 14.1203 | 0.1611 | 87.64 | <.0001 | 1.40E+06 | 9.90E+05 | 1.90E+06 |
| GLO | 13.8100 | 0 | _ | _ | 9.90E+05 | 9.90E+05 | 9.90E+05 |
| BUN | -0.0651 | 0.0813 | -0.80 | 0.4237 | 0.94 | 0.80 | 1.10 |
| SCr | 0.0155 | 0.0096 | 1.62 | 0.1045 | 1.02 | 1 | 1.03 |
| TC | 0.2706 | 0.0905 | 2.99 | 0.0028 | 1.31 | 1.1 | 1.57 |
| Hb | 0.259 | 0.1446 | 1.79 | 0.0732 | 1.30 | 0.98 | 1.72 |
| HCT | -0.8245 | 0.4875 | -1.69 | 0.0908 | 0.44 | 0.17 | 1.14 |
| MCV | 0.5528 | 0.3794 | 1.46 | 0.1451 | 1.74 | 0.83 | 3.66 |
| MCH | -1.6046 | 1.125 | -1.43 | 0.1538 | 0.20 | 0.02 | 1.82 |
| MPV | -1.1376 | 0.7905 | -1.44 | 0.1501 | 0.32 | 0.07 | 1.51 |
