# Supplementary material for: Association between Leukocyte and Metabolic Syndrome in Urban Han Chinese: A Longitudinal Cohort Study
Source: PLoS One. 2012 Nov 27;7(11):e49875. doi: 10.1371/journal.pone.0049875 (PMC3507923; doi:10.1371/journal.pone.0049875)
Supplement: Table S12 — Multiple GEE analysis of leukocyte and hyperglycemia after adjusting potential confounding factors. (DOC) [file pone.0049875.s012.doc]

**Table S12 Multiple GEE analysis of leukocyte and hyperglycemia after** adjusting potential confounding factors

| **Variable** | **Estimate** | **Error** | **Z** | **Pr>|Z|** | **RR** | **Lower 95% confidence limits** | **Upper 95% confidence limits** |
| --- | --- | --- | --- | --- | --- | --- | --- |
| **leukocyte** |  |  |  |  |  |  |  |
| Q4 | 0.5488 | 0.1044 | 5.25 | <0.0001 | 1.7312 | 1.4107 | 2.1244 |
| Q3 | 0.2681 | 0.1063 | 2.52 | 0.0116 | 1.3075 | 1.0616 | 1.6101 |
| Q2 | -0.0188 | 0.1116 | -0.17 | 0.8661 | 0.9814 | 0.7885 | 1.2213 |
| Q1 | ref | ref | ref | ref | ref | 1 | 1 |
| age | 0.0002 | 0.0038 | 0.07 | 0.9482 | 1.0002 | 0.9928 | 1.0077 |
| gender | -0.2259 | 0.1302 | -1.73 | 0.0828 | 0.7978 | 0.618 | 1.0297 |
| time | 0.3939 | 0.0253 | 15.58 | <0.0001 | 1.4828 | 1.4111 | 1.558 |
| GGT | 0.0067 | 0.0013 | 5.3 | <0.0001 | 1.0067 | 1.0042 | 1.0091 |
| ALB | -0.0161 | 0.045 | -0.36 | 0.7202 | 0.984 | 0.901 | 1.0747 |
| GLO | 0.0088 | 0.057 | 0.15 | 0.877 | 1.0088 | 0.9022 | 1.1282 |
| BUN | 0.0912 | 0.0337 | 2.71 | 0.0068 | 1.0955 | 1.0255 | 1.1701 |
| SCr | 0.0006 | 0.0023 | 0.28 | 0.779 | 1.0006 | 0.9962 | 1.0051 |
| TC | 0.2717 | 0.0412 | 6.6 | <0.0001 | 1.3122 | 1.2105 | 1.4225 |
| HB | 0.0017 | 0.0044 | 0.39 | 0.6955 | 1.0017 | 0.9931 | 1.0105 |
| HCT | -0.0032 | 0.0222 | -0.15 | 0.8846 | 0.9968 | 0.9544 | 1.0411 |
| diet | 0.0535 | 0.0401 | 1.33 | 0.1819 | 1.055 | 0.9753 | 1.1411 |
