# Supplementary material for: Association between Leukocyte and Metabolic Syndrome in Urban Han Chinese: A Longitudinal Cohort Study
Source: PLoS One. 2012 Nov 27;7(11):e49875. doi: 10.1371/journal.pone.0049875 (PMC3507923; doi:10.1371/journal.pone.0049875)
Supplement: Table S13 — Multiple GEE analysis of leukocyte subtypes and hyperglycemia after adjusting other potential confounding factors. (DOCX) [file pone.0049875.s013.docx]

**Table S13 Multiple GEE analysis of leukocyte subtypes and hyperglycemia after adjusting other potential confounding factors**

| **Variable** | **Estimate** | **Error** | **Z** | **Pr>\|Z\|** | **RR** | **Lower 95% confidence limit** | **Upper 95% confidence limits** |
| --- | --- | --- | --- | --- | --- | --- | --- |
| **lymphcyte** |  |  |  |  |  |  |  |
| Q4 | 0.1759 | 0.1126 | 1.56 | 0.1181 | 1.1923 | 0.9563 | 1.4866 |
| Q3 | -0.1950 | 0.1134 | -1.72 | 0.0854 | 0.8228 | 0.6588 | 1.0276 |
| Q2 | -0.1699 | 0.1092 | -1.56 | 0.1197 | 0.8437 | 0.6811 | 1.0451 |
| Q1 | ref | ref | ref | ref | ref | 1 | 1 |
| **monocyte** |  |  |  |  |  |  |  |
| Q4 | 0.0571 | 0.1261 | 0.45 | 0.6507 | 1.0588 | 0.8269 | 1.3557 |
| Q3 | -0.0014 | 0.1192 | -0.01 | 0.9903 | 0.9986 | 0.7905 | 1.2614 |
| Q2 | 0.0623 | 0.1102 | 0.57 | 0.5720 | 1.0643 | 0.8575 | 1.3209 |
| Q1 | ref | ref | ref | ref | ref | 1 | 1 |
| **neutrophil** |  |  |  |  |  |  |  |
| Q4 | 0.5180 | 0.1102 | 4.70 | <0.0001 | 1.6787 | 1.3526 | 2.0832 |
| Q3 | 0.3314 | 0.1062 | 3.12 | 0.0018 | 1.3929 | 1.1312 | 1.7151 |
| Q2 | 0.0417 | 0.1094 | 0.38 | 0.7034 | 1.0426 | 0.8413 | 1.2919 |
| Q1 | ref | ref | ref | ref | ref | 1 | 1 |
| age | 0.0003 | 0.0038 | 0.08 | 0.9337 | 1.0003 | 0.9929 | 1.0078 |
| gender | -0.2311 | 0.1307 | -1.77 | 0.0771 | 0.7937 | 0.6142 | 1.0254 |
| time | 0.3947 | 0.0261 | 15.15 | <0.0001 | 1.4839 | 1.4100 | 1.5617 |
| GGT | 0.0069 | 0.0013 | 5.41 | <0.0001 | 1.0069 | 1.0044 | 1.0094 |
| ALB | -0.0184 | 0.0447 | -0.41 | 0.6809 | 0.9818 | 0.8993 | 1.0718 |
| GLO | 0.0060 | 0.0567 | 0.11 | 0.9156 | 1.0060 | 0.9002 | 1.1242 |
| BUN | 0.0900 | 0.0337 | 2.67 | 0.0076 | 1.0942 | 1.0243 | 1.1688 |
| SCr | 0.0006 | 0.0023 | 0.25 | 0.8014 | 1.0006 | 0.9961 | 1.0051 |
| TC | 0.2702 | 0.0412 | 6.56 | <0.0001 | 1.3102 | 1.2085 | 1.4203 |
| HB | 0.0011 | 0.0044 | 0.25 | 0.8062 | 1.0011 | 0.9924 | 1.0098 |
| HCT | -0.0011 | 0.0221 | -0.05 | 0.9612 | 0.9989 | 0.9566 | 1.0431 |
| diet | 0.0577 | 0.0401 | 1.44 | 0.1506 | 1.0594 | 0.9792 | 1.1460 |
| smoking | -0.0272 | 0.0245 | -1.11 | 0.2673 | 0.9732 | 0.9276 | 1.0210 |
